# The Wear Behavior of the Laser Cladded Ti-Al-Si Composite Coatings on Ti-6Al-4V Alloy with Additional TiC

**DOI:** 10.3390/ma14164567

**Published:** 2021-08-14

**Authors:** Ran Liu, Xianting Dang, Yuan Gao, Tao Wu, Yuanzhi Zhu

**Affiliations:** 1 Department of Materials Science and Engineering, School of Mechanical and Materials Engineering, North China University of Technology, 5 Jinyuanzhuang Road, Beijing 100144, China; dangxianting@ncut.edu.cn (X.D.); gaoyuan@ncut.edu.cn (Y.G.); tozyz1@163.com (Y.Z.); 2 Department of Materials Technology, Zhejiang Institute of Mechanical & Electrical Engineering, 528 Binwen Road, Hangzhou 310053, China; wutao2@zime.edu.cn

**Keywords:** titanium alloy, Ti-Al-Si + *x*TiC composite, microstructure, wear resistance, laser cladding

## Abstract

In this study, the Ti-Al-Si + *x*TiC (*x* = 0, 2, 6, 10 wt.%) composite coatings, each with a different content of TiC were fabricated on a Ti-6Al-4V alloy by laser surface cladding. The microstructure of the prepared coatings was analyzed by the scanning electron microscopy (SEM), energy dispersive spectroscopy (EDS), and X-ray diffraction (XRD). The microhardness and the wear resistance of these coatings were also evaluated. The results show that α-Ti, Ti_3_Al, Ti_5_Si_3_, TiAl_3_, TiAl, Ti_3_AlC_2_ and TiC particles can be found in the composites. The microstructure can obviously be refined by increasing the content of TiC particles, while the microhardness increases and the coefficient of friction decreases. The Ti-Al-Si-6TiC composite shows the best wear resistance, owing to its relatively fine microstructure and high content of TiC particles. The microhardness of this coating is 5.3 times that of the substrate, while the wear rate is only 0.43 times. However, when the content of TiC was up to 10 wt.%, the original TiC could not be dissolved completely during the laser cladding process, leading to formation of cracks on the coatings.

## 1. Introduction

Titanium and its alloy have extensive usage in aerospace, chemical processing, power generation, marine, sports, medical and transportation industries by virtue of their low density, high specific strength, high creep resistance, high temperature mechanical properties, and superior corrosion resistance [1,2]. If these alloys are to be used in a wider application area, such as in load bearing contacts, then wear resistance improvement is necessary. The tribological performance of titanium alloys are often poor due to their low resistance to plastic shearing and low strain hardening [3], and surface modification is often used to improve the surface wear resistance of titanium alloys at room temperature [4,5,6,7,8].

Generally, surface modification techniques consist of two major aspects: one is processing technology, and the other is the design of coating material. In recent years, many researchers have improved the wear resistance, high temperature oxidation resistance and corrosion resistance of titanium alloy through a series of surface modification technologies, such as ion infiltration [4], surface nitriding [5], carburizing [6], spraying [7], and surface laser treatment [2,8]. Among them, laser cladding is an effective surface modification technology, which is easy to control and can obtain a surface with good metallurgical bonding [8].

Metal matrix ceramic composites such as Co-base [9,10], Ni-base [11] and Ti-base materials [1,4,5] are commonly used material systems fabricated by laser cladding. The metal or alloy in the composite can improve the wettability between ceramic phase and matrix, and reduce the residual stress and cracking tendency. Compared with the Ti-6Al-4V substrate, the microhardness of the coating can usually be increased by 3–6 times, and the wear rate can also be reduced to 0.15–0.3 [1,5,9,10,11].

To further improve the room temperature surface wear resistance of titanium alloys, particulate reinforcements, such as metal oxides nitrides [12], borides [13,14], and carbides [15,16,17] are widely used in the wear resistant materials. Among them, TiC exhibits both excellent high temperature abrasive and corrosion resistance because of its high hardness, unique strong atomic bond, and high stability under high temperature exposure, which makes it suitable to be used as coating reinforcement. Li et al. [15] employed Al + TiC powders to fabricate TiC reinforced Ti-based composite coatings by laser cladding, while Yang et al. [18] fabricated TiCN/Ti composite coatings on Ti-6Al-4V alloy. In Xu et al.’s work [19], TiC particles was added into Ti-Fe powders to fabricate composite coating using spark plasma sintering. The above results revealed that the microhardness and surface wear resistance of titanium alloys were improved after employing TiC particles as reinforcements in the composite coatings.

In our previous work, various Al/Si ratios of Ti-Al-Si composite coatings were prepared [20]. The results showed that the hardness and wear resistance of Ti-Al-Si coating can be significantly improved compared to the Ti-6Al-4V substrate. Thus, the aim of this paper is to develop an excellent wear composite coating based on the Ti-Al-Si coating of Ti-6Al-4V substrate by laser cladding using mixtures of Ti-Al-Si-TiC as the precursor. Microhardness and wear resistance properties were conducted to evaluate the coatings properties. The microstructure of the cladded zone was characterized using a scanning electron microscope (SEM) and energy dispersive spectrometer (EDS).

## 2. Experimental Procedure

### 2.1. Materials Preparation

In the present study, Ti-6Al-4V alloy samples with dimensions of 100 mm× 15 mm × 6 mm were used as the substrate. The surface treatments of the raw specimens included grinding down to a final 800# SiC grit paper and degreasing in ethanol ultrasonically for 10 min to remove the impurities and oxide layer before the laser cladding operation. Ti-Al-Si-*x*TiC (*x* = 0, 2, 6, 10, wt.%) composites were prepared by ultra-fined Ti (purity > 99.9%), Al (purity > 99.9%), Si (purity > 99.9%), and TiC (purity > 99.8%) powders, which were supplied by Jinan Institute of Metallurgical Science.

In order to make the mixed powders more homogeneous, the mixture was milled via ball milling for 2 h and the milling speed was 150 r/min. To obtain powders with good fluidity, the evenly mixed powders were dried at 343 K in a dryer for 2 h. Meanwhile, the water glass solution of Na_2_O·nSiO_2_ to deionized water was prepared with a volume ratio of 1:3. The water glass solution was dropped into the mixed powders, about 10 drops for 1 g of powders, before being fully stirred with a glass rod. Then the powders were put into a self-made mold on the surface of the substrate and the preset thickness was about 0.8 mm. The compositions of Ti-Al-Si based composites are listed in Table 1. For simplicity, the composites are named as Ti-Al-Si, Ti-Al-Si-2TiC, Ti-Al-Si-6TiC, and Ti-Al-Si-10TiC according to the content of added TiC.

YLR-1000 laser was used in laser cladding experiment and scanned with single layer single channel. The parameters of laser cladding process: laser power 600 W, scanning speed 15 mm/min and laser beam diameter 4 mm. After laser cladding, the specimens were cut into 10 mm × 10 mm × 6 mm cube using the wire electrical discharge machining (WEDM) technology in a cross section perpendicular to the cladding direction.

### 2.2. Testing and Characterization

The microstructure of the powders and coatings were observed using field emission scanning electron microscope (SEM, ZEISS Sigma-300, Zeiss, Oberkochen, Germany) and combined with energy dispersive spectrometry (EDS, Bruker, Billerica, MA, USA) to analyze the distribution of elements. The specimens were polished and etched in a solution of HF:HNO_3_:H_2_O = 1:1:10 (volume ratio) to reveal the microstructure of the alloyed layer. Compounds formed in the laser-alloyed layers were investigated using physics X-ray diffraction (Ultima IV, Rigaku, Tokyo, Japan) with Cu K_α_ radiation operated at a voltage of 40 kV at room temperature, a tube current of 40 mA, and a scanning rate of 10°/min.

The microhardness of substrate and coatings with different content of TiC were measured by FM-810 digital Vicker’s hardness tester with the load of 100 g and the dwell time of 10 s. The hardness was measured every 100 μm from the cladding surface to the substrate. Tribological tests were performed using CFT-I wear test machine with a load of 980 g at room temperature. A sintered GCr15 steel ball with a diameter of 5 mm was selected as counterpart. The test duration was 30 min with the reciprocating speed n of 200 r/min and a wear mark length of 5 mm. The coefficient of friction (COF) was recorded automatically. The worn morphologies of the samples were analyzed using SEM to show the worn mechanism. The mass loss of the sample before and after wear were weighed by an electronic balance. Then, according to classical wear equation, the wear rate ω can be calculated by the following equation:

Ω = Δ*m*/(π*dn*) (1)

where ω is the wear rate, Δ*m* is the wear loss quality (g), *n* is the rotating speed (r/min), *d* is the sliding distance (*m*).

## 3. Results and Discussion

### 3.1. Morphologies and XRD Analysis of Powders

**Figure 1:**
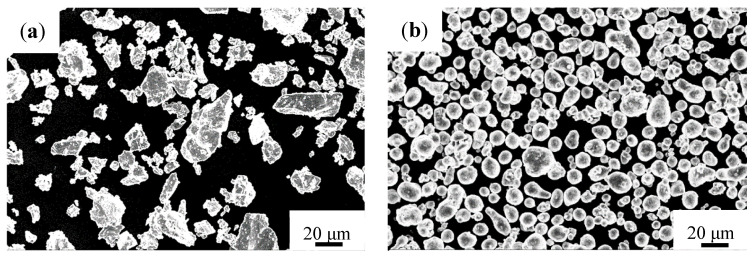

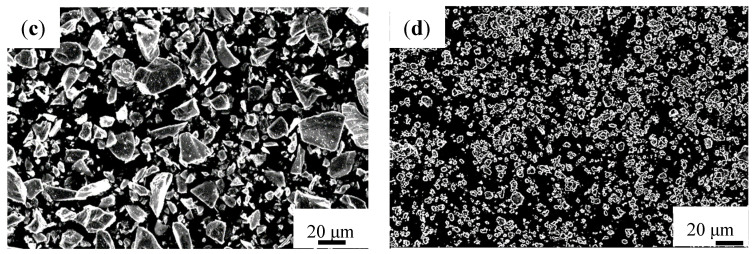
Morphologies of powders (**a**) Ti; (**b**) Al; (**c**) Si and (**d**) TiC.

Figure 1 shows the morphologies of as-received Ti, Al, Si and TiC powders, respectively. Ti and Si powders have irregular shapes with a mean particle size of 32.4 and 21.9 μm, respectively. Aluminum powders have a relatively uniform spherical shape, with average particle size of about 14.9 μm, while those found in the TiC powders are smaller than 7 μm.

**Figure 2:**
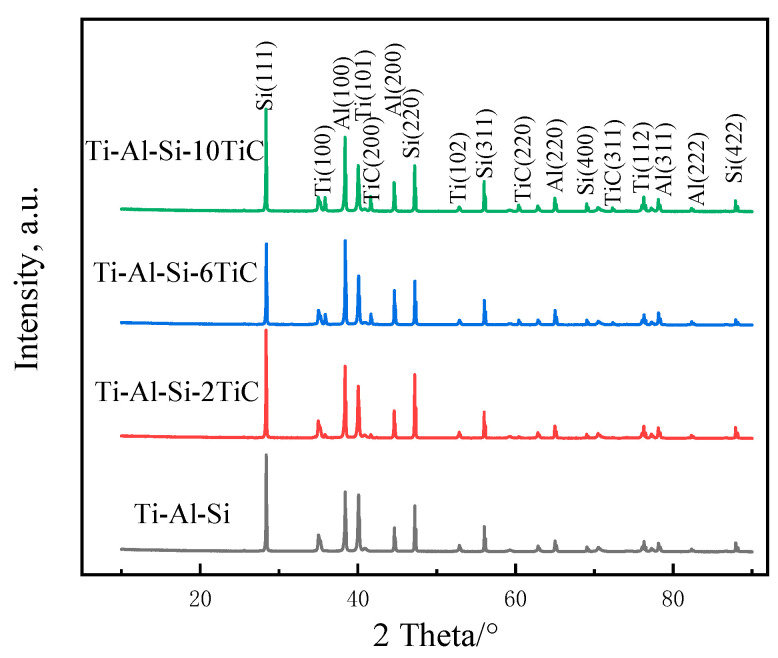
XRD patterns of Ti-Al-Si-*x*TiC powders after mechanical mixing.

To compare with the generated phases in composite coating, the XRD patterns of the as-milled Ti-Al-Si based powders are shown in Figure 2. The index of XRD peaks suggests that hcp-Ti, fcc-Al and diamond structure Si exist in all laser cladding powders. The weak diffraction peaks of TiC only present at about 42° in the XRD pattern of Ti-Al-Si-2TiC, owing to the low content of TiC (only 2 wt.%). However, the diffraction peaks of TiC can be clearly detected in the XRD patterns of Ti-Al-Si-6TiC and Ti-Al-Si-10TiC powders, as shown in 42°, 61°, and 73°.

### 3.2. Morphologies of the Composites Coatings

**Figure 3:**
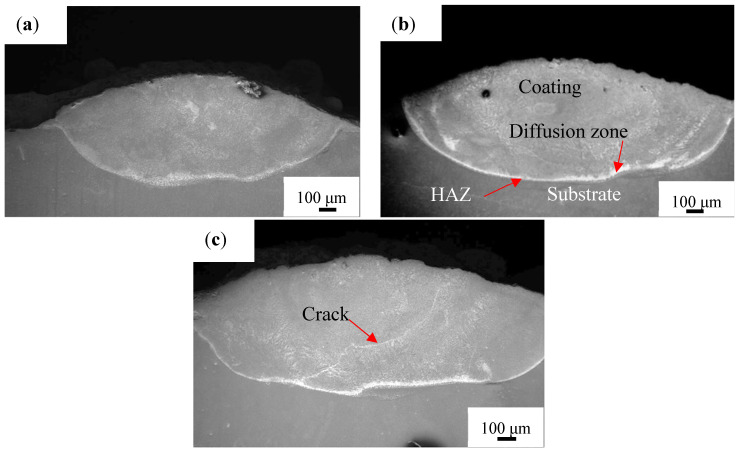
The overall morphology of coatings on the cross section. (**a**) Ti-Al-Si, (**b**) Ti-Al-Si-6TiC and (**c**) Ti-Al-Si-10TiC.

Figure 3 shows the overall morphologies of the coatings on the cross section. No obvious cracks or pores are observed in the Ti-Al-Si and Ti-Al-Si-6TiC coatings, but cracks appeared in the coating containing 10% TiC, as shown in Figure 3c, which also shows that the cladding layer can be divided into four zones: the coating, diffusion, and the heat affected zone (HAZ) as well as the substrate.

**Figure 4:**
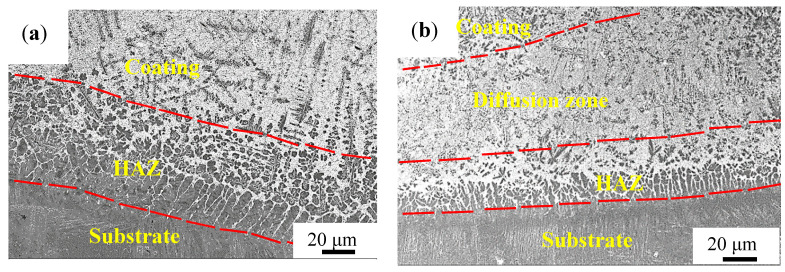

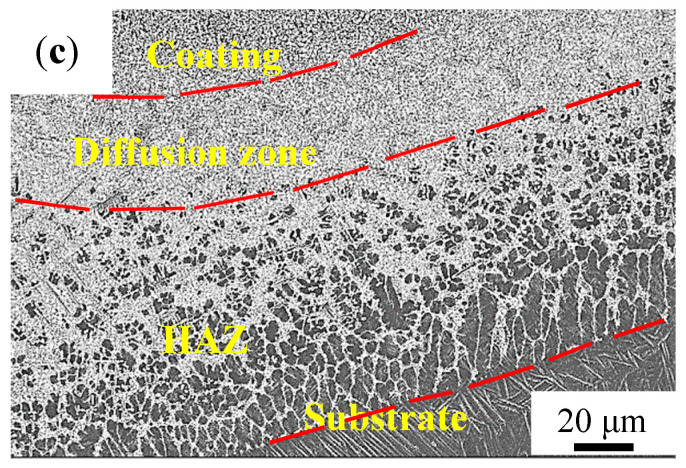
Micrographs at the bottom of the coatings on the cross section. (**a**) Ti-Al-Si, (**b**) Ti-Al-Si-6TiC and (**c**) Ti-Al-Si-10TiC.

The morphologies at the bottom of the coating are shown in Figure 4. In the Ti-Al-Si coating, the microstructure of the cladding layer is sparse, and the diffusion zone is not obvious. In the coatings with TiC, the reinforcing phase TiC is scattered in the cladding layer and has no obvious gradient distribution characteristics. The structure is well developed with obviously denser and finer crystals. In the heat affected zone, the coating and the substrate are intertwined. This is because the two zones have the same structure, which are both needle-shaped α-Ti with good compatibility. The similar needle-shape structure is shown in Wu et al.’s work, where it is inferred that it is a saturated acicular structure of α-Ti solid solution containing Al, V, Si, and other elements [21]. Therefore, they can be closely fused with each other to form a good metallurgical bonding, and it can be inferred that the coating and the substrate have a high bonding strength, which is beneficial to improve the service life of the coating.

**Figure 5:**
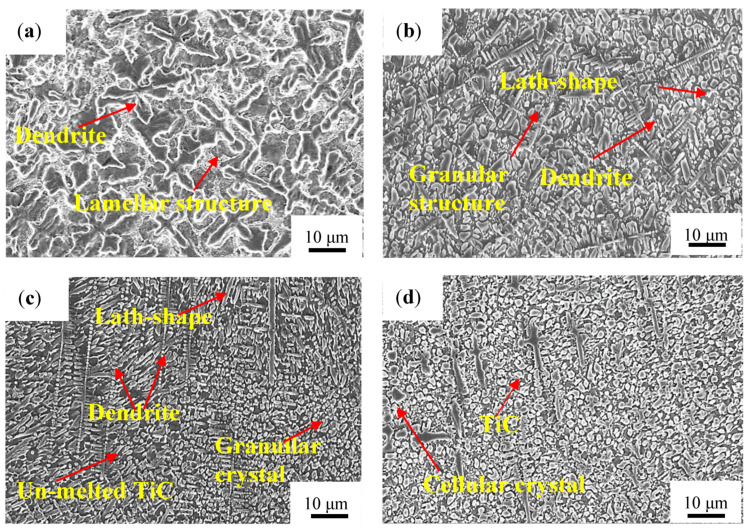
Morphologies at the middle of coatings on the cross section. (**a**) Ti-Al-Si, (**b**) Ti-Al-Si-2TiC, (**c**) Ti-Al-Si-6TiC and (**d**) Ti-Al-Si-10TiC.

Figure 5 shows the typical microstructures of the four coatings, respectively, at the middle zone of the coatings. It is worth noting that there is a significant difference in the cross-sectional microstructure of the coating along the depth direction. As can be seen from Figure 5a, dendrites and surrounded lamellar crystals are generated in the Ti-Al-Si laser cladding layer because of the high supercooling. Therefore, the crystals of the coating are coarse and the dendrites do not show obvious preferred orientation.

The coating structure with TiC is mainly composed of lamellar crystals, granular crystals, cellular crystals, and dendrites, while the layer-structured eutectic structure almost disappears. In the Ti-Al-Si-2TiC coating, TiC decomposed first and then precipitated and grew in the form of dendrite. In fact, after laser cladding, TiC was the nucleus in the dendrite. While when the TiC content is high, the reinforcing phase TiC is mainly in the form of particles, granular TiC gathers next to the rod-shaped crystal, growing according to a certain rule, and the dendrite arms in the cladding layer are finer. The occurrence of un-melted particles of TiC is due to the faster cooling rate of the molten pool during the laser cladding process, which results in a short existence of the molten pool, making the bulk of TiC unable to melt, meaning it is retained during the solidification process. As the mass fraction of TiC in the coating powder increases, the fraction of dendrites in the sample decrease, and the crystal structure is significantly refined compared with the structure without TiC, and the coating structure of Ti-Al-Si-6TiC is the finest. This might be due to the formation of Al-Ti-C during the crystallization process. Studies have shown that TiC can be used as the crystalline core of Al-Ti-C which is a very good grain refining agent which has an obvious refining effect [22].

The microstructure of the Ti-Al-Si-10TiC coating is relatively uniform, comprised of dendrites and equiaxed cellular crystals. Research by Ding et al. [23] showed that the refining effect of Ti-Al-C grain refiner was due to the precipitation of TiC particles. Meanwhile, the thermal physics coefficient between TiC and the matrix is very different. This led to the generation of thermal stress, resulting in cracks, which makes the cladding less efficient. The same results have been found in Xu et al.’s study [19], where it was found that the microstructure of Ti-Fe was obviously refined with increasing the content of TiC particles.

### 3.3. XRD and EDS Analysis of Coatings

**Figure 6:**
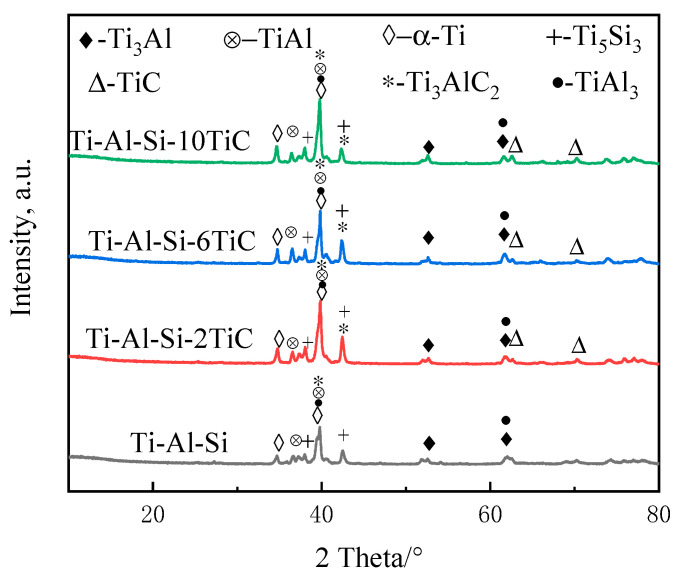
XRD patterns of Ti-Al-Si-*x*TiC coating after laser cladding.

The XRD patterns in Figure 6 show the phase constitution of the laser cladding Ti-Al-Si + *x*TiC coatings. The results indicate that there are α-Ti, Ti_3_Al, TiAl, TiAl_3_ and Ti_5_Si_3_ phases in the Ti-Al-Si laser cladding layer. These phases were beneficial to improve the wear resistance of the coating compared to the Ti-6Al-4V substrate [10].

As shown in the XRD patterns of the mixed powders in Figure 2, no TiC phase is found in Ti-Al-Si coatings. The Gibbs free energy ΔG of TiAl, Ti_3_Al, TiAl_3_ and Ti_5_Si_3_ phases, which were generated in situ, have been consulted using the thermodynamic date from Ref. [24]. The ΔG of the above phases are all negative, in which the ΔG of Ti_5_Si_3_ phase is the smallest of all, namely Ti_5_Si_3_ forming in priority throughout the temperature range, and where Ti_5_Si_3_ is a compound with high thermal stability. It can be inferred that this phase occupies a higher fraction of the coating. Therefore, the peaks at 42° should be Ti_5_Si_3_ phases, which is consistent with Wu’s study [20].

The peak at 42° in the Ti-Al-Si-*x*TiC coating disappears, while the 62° and 73° peaks in the Ti-Al-Si-6TiC and Ti-Al-Si-10TiC coatings still exist, indicating that part of TiC decomposed during laser processing. Meanwhile, TiC phase generated accompanied by the formation of Ti_3_AlC_2_ ceramic phase together with Al, and there is still a part of TiC that is not decomposed. The Ti_3_AlC_2_ in situ formation can also improve the mechanical properties of the coatings, since the Ti_3_AlC_2_ has both properties of ceramic and metallic materials [23]. However, the fraction of Ti_3_AlC_2_ phase is not very high, which may be caused by the relatively small mass fraction of the C element. Meanwhile, the mass fractions of the three elements Ti, Al, and Si are high, which can react quickly to form a variety of compounds. It was also found that the intensity of the TiC phase increased with the increasing mass fraction of the TiC powder. In addition, the intensity of the Ti_3_Al, TiAl, TiAl_3_ and Ti_5_Si_3_ phases changed with the increasing mass fraction of the TiC powder.

**Figure 7:**
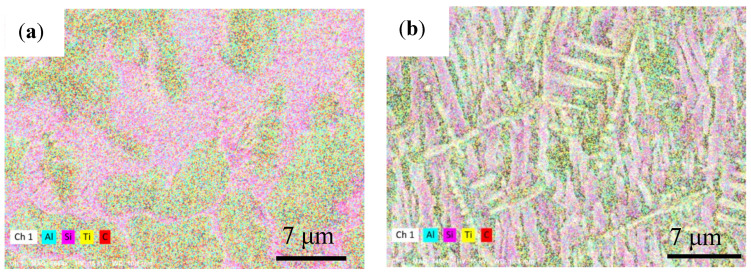

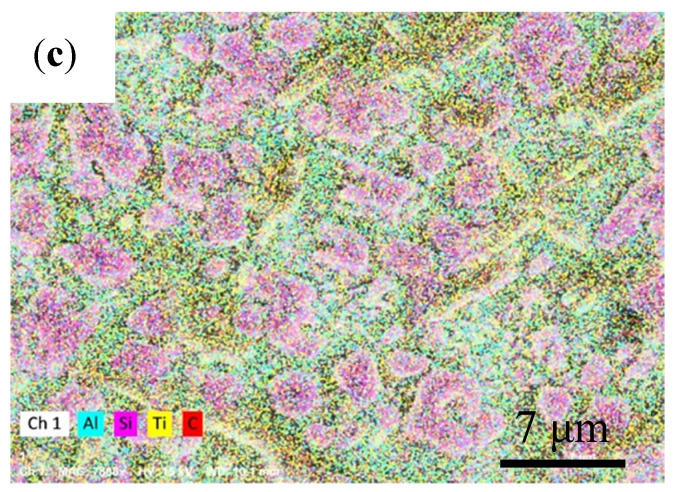
EDS mapping of Ti-Al-Si-6TiC composite showing distribution of elements at the (**a**) interface, (**b**) middle and (**c**) upper zones.

In order to further determine the distribution of elements at different zones of the coating, energy dispersive spectroscopy (EDS) was utilized, and the results are as shown in Figure 7. It is shown that the distribution of Si element shows the most obvious change, which is mainly distributed in the lamellar structure at the bottom, the dendrite in the middle, and the cellular structure at the upper part in the form of α-Ti/Ti_5_Si_3_ eutectic structure. Al is concentrated around Si, meanwhile Ti is evenly distributed everywhere due to its high proportion, and it can be concluded that the matrix of the coating is mainly Ti-Al intermetallic compound. Although the distribution of C element at the upper and interface junction is dispersed, it is still mainly located between lamellar crystals and dendrites, and a small amount of granular TiC crystals exist. As the solidification temperature decreases, the molten pool gradually solidifies. When the temperature drops below the liquidus line, TiC crystals begin to precipitate. As the cooling rate of TiC in the molten pool is relatively fast, TiC grows in a dendritic form. Due to the high cooling rate, the growth rate of TiC is less than the growth of TiC nucleation rate, and the TiC crystals shown are relatively small.

### 3.4. Microhardness Analysis

**Figure 8:**
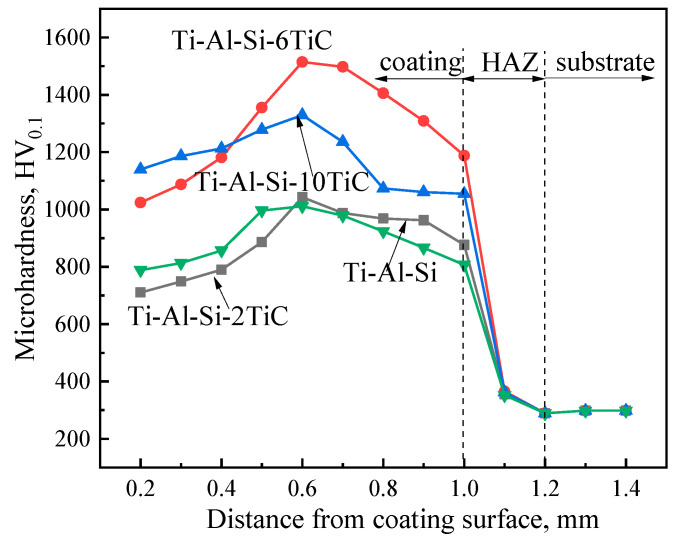
Microhardness curves from the surface of the coating to substrate.

The microhardness distribution from the cladding surface to the substrate is shown in Figure 8. It is shown that the microhardness distribution trends of all cladding layers are similar. The microhardness distribution of the coatings with different contents of TiC can be divided into three-stage which is consistent with the microstructure. The hardness of the dilution zone is significantly lower than that of the cladding zone, which is caused by the dilution effect of the substrate on the coating. The area within 1.0 mm from the surface has a higher microhardness and is a laser cladding coating layer. The microhardness of the cladding layers of each sample is significantly higher than that of the substrate. The 1.0–1.2 mm area from the surface is the heat affected zone (HAZ), and its microhardness drops rapidly. The third zone is substrate from 1.2 mm inside with an average microhardness of 285 HV_0.1_.

It is shown in the Figure 8 that the hardness of the coating with 2 wt.% TiC is basically the same as that of the coating without TiC, which may be because the TiC content is too small and does not have a strengthening effect. The maximum microhardness of the coating with 2 wt.% TiC and without TiC are 1042 and 1011 HV_0.1_, respectively, which are 3.6 and 3.5 times of the substrate. The coating containing 6 wt.% TiC increases significantly from the surface to the coating, and the hardness is the highest at 600 μm from the surface, which is 1515 HV_0.1_, 5.3 times of the substrate and 1.5 times of the Ti-Al-Si coating, before the microhardness gradually decreases during the extension to the substrate. The microhardness of Ti-Al-Si-10TiC coating gradually increases from 0 to 600 μm, and reaches a maximum of 1325 HV_0.1_ before gradually decreasing. According to the analysis above, the resulting microstructure is finer and the fine-grain strengthening effect is more pronounced. In addition, the coatings contain hard phases like Ti_5_Si_3_, TiAl, Ti_3_Al and TiC can significantly improve the surface hardness of the cladding layer [25]. Moreover, the hardness of the coating containing 10% TiC decreases due to the coarsening of its microstructure compared with the coating containing 6% TiC.

### 3.5. Friction and Wear Testing Analysis

**Figure 9:**
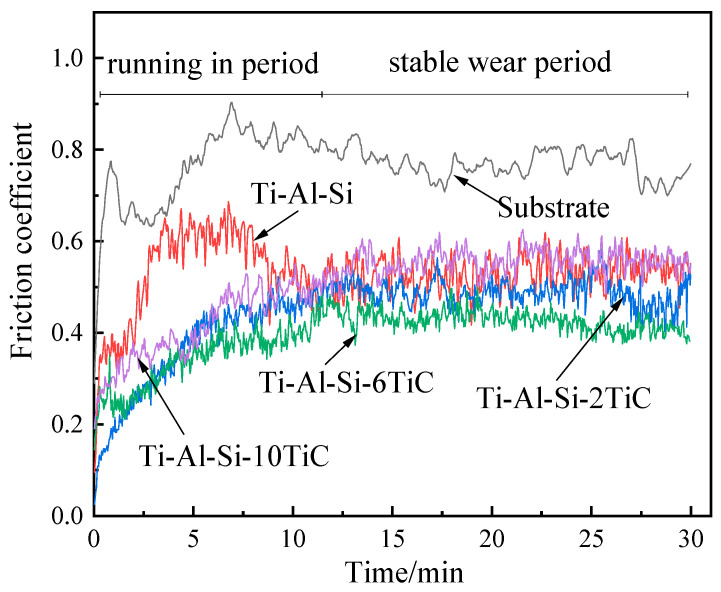
Typical evolution of friction coefficients of Ti-Al-Si-*x*TiC composites with sliding time.

Friction and wear tests were carried out on the substrate and several laser claddings layers separately for 30 min. The coefficient of friction (COF) curves of the Ti-Al-Si-*x*TiC (*x* = 0, 2, 6, 10) composites are shown in Figure 9. It is shown that the substrate and Ti-Al-Si composite process a high average COF value of 0.77 and 0.51. The addition of TiC particles can significantly decrease the COF value of Ti-Al-Si based composite. The COF values of the Ti-Al-Si-2TiC, Ti-Al-Si-6TiC, Ti-Al-Si-10TiC composites are 0.50, 0.39 and 0.44, respectively. The obvious decrease of COF value with the addition of TiC particles can be explained by the improved microhardness which prevents the penetrating effect of the indenter.

**Figure 10:**
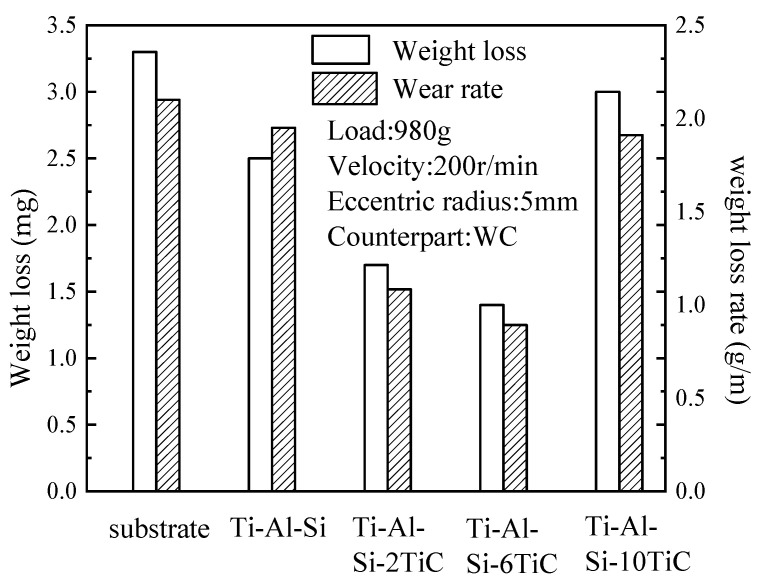
Mass loss and wear rate of substrate and the laser cladding coatings.

Figure 10 shows the data of the wear loss for the composite coatings and substrates. The results reveal that the wear mass loss and wear rate of the Ti-6Al-4V substrate are the highest, namely, 3.3 mg and 2.1 g/m, respectively. The wear mass loss of the Ti-Al-Si coating without TiC is 2.5 mg, while the wear mass of the coating with 6% TiC is the smallest at 1.4 mg, which is mainly attributed to their higher microhardness than that of the Ti-6Al-4V alloy. The wear loss of the coating with TiC is less than the wear amount of the Ti-Al-Si coating without TiC. Similarly to the microhardness results, the wear resistance of the Ti-Al-Si-10TiC coating is slightly lower compared to Ti-Al-Si-6TiC when reduced. Too much TiC will reduce the wear resistance of the coating, which is mainly due to the high TiC content that reduces the plasticizing effect of the coating. The tendency will produce a higher cracking phenomenon, and under the action of the GCr15 steel ball in the cladding, cracks will continue to form and expand; moreover, because of the falling of wear debris, the overall wear loss will increase significantly. The similar phenomenon is also reported in Ti-Fe-TiC composite [16]. Therefore, the hardness and wear resistance of the cladding coatings can be greatly improved when the concentration of TiC is higher and the grain size is smaller.

**Figure 11:**
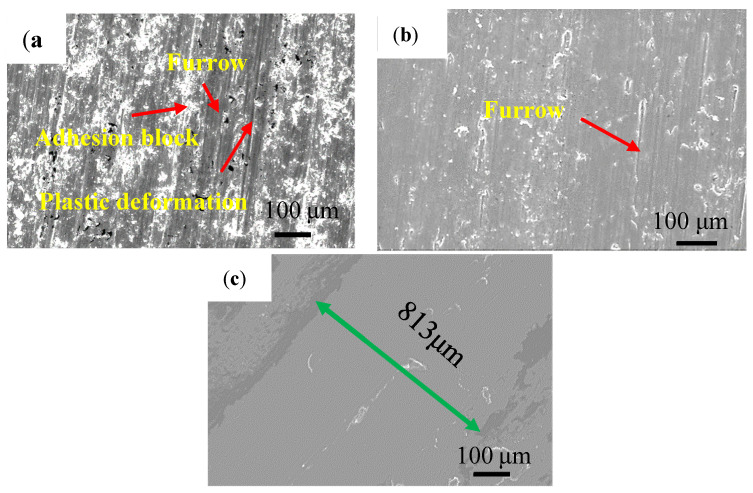
Morphologies of worn track of (**a**) Ti-6Al-4V substrate; (**b**) Ti-Al-Si; (**c**) Ti-Al-Si-6TiC.

The worn morphologies with different content of TiC powder are shown in Figure 11. Under the sliding wear conditions with quenching and tempering steels (GCr15) as the counterpart, the hard asperities on the surface can easily penetrate the sliding surface of the Ti-6Al-4V alloy since the microhardness of the Ti-6Al-4V alloy (about 285 HV) was much lower than that of the counterpart (about 800 HV); thus, the deep grooves and the adhesive features were present in its worn surface are shown in Figure 11a. Serious plastic deformations on the worn surface of the Ti-6Al-4V substrate were observed, with varying degrees of furrows and scaly wear debris, indicating that the wear mechanism of the substrate is adhesive wear.

The worn morphology of the Ti-Al-Si layer is much smoother than the substrate, as seen in Figure 11b. The surface of the sample is distributed with scratches consistent with the sliding direction and dispersed white particles. The wear quality is 0.77 that of the titanium alloy substrate under the same experimental conditions, indicating that the existence of reinforcement phases has improved the wear resistance of titanium alloy matrix. In addition, there is no obvious plastic deformation on the worn surface of the Ti-Al-Si coating, as only a small amount of white peeling and pits formed after peeling. This may be due to the higher hardness of the coating substrate, which can reduce the plastic deformation caused by adhesive wear. The Ti_5_Si_3_ phase, which is distributed in a network, and other hard phases, bear most of the forces involved in wear.

Figure 11c shows the wear morphology of the coating containing 6 wt.% TiC. The structure of the cladding layer is refined, and the surface hardness is relatively high. Under the action of the friction wheel, the wear morphology is relatively smooth. In addition, there are many hard phases, such as TiC, in the cladding layer, which block and limit the development of wear marks. The furrow is shallow and fine, and its wear mechanism is abrasive wear. Friction and wear properties are the result of multiple actions such as refinement of microstructure and the reinforcement of hard phases. Compared with the substrate, the wear mechanism of the coating is mainly abrasive wear, and adhesive wear is a supplement. At the same time, the presence of high hardness TiC means it plays a role in the hard skeleton support of the cladding layer, thereby reducing the wear rate effectively.

## 4. Conclusions

The Ti-Al-Si-*x*TiC composite was coated on the Ti-6Al-4V alloy to enhance its wear resistance by the laser cladding technology. The following conclusions can be drawn:

(1) Ti_3_AlC_2_ particles were formed in the laser cladding process, which strengthened the formed coatings.
(2) When the content of TiC was higher than 6 wt.%, the original TiC could not be dissolved completely in the laser cladding process, resulting in the cracks in the formed coatings.
(3) The laser cladded Ti-Al-Si−6TiC composite coatings have the highest wear resistance of the formed Ti-Al-Si−*x*TiC coatings.

## Figures and Tables

**Table 1:** Compositions of Ti-Al-Si-*x*TiC composites (wt.%).

Composite	Ti	Al	Si	TiC
Ti-Al-Si	50	30	20	0
Ti-Al-Si-2TiC	48	30	20	2
Ti-Al-Si-6TiC	44	30	20	6
Ti-Al-Si-10TiC	40	30	20	10

## Data Availability

The data presented in this study are available on request from the corresponding author.
